# The prevalence and factors associated for anti-tuberculosis treatment non-adherence among pulmonary tuberculosis patients in public health care facilities in South Ethiopia: a cross-sectional study

**DOI:** 10.1186/s12889-017-4188-9

**Published:** 2017-03-20

**Authors:** Tadele Teshome Woimo, Wondwossen Kassahun Yimer, Temesgen Bati, Hailay Abrha Gesesew

**Affiliations:** 1 Department of Infectious diseases, Dawro District Health Office, Dawro, Ethiopia; 2 US Department of Human and Health Services, Biostatistics and Bioinformatics Branch (BBB), NICHD, New York, USA; 3 Department of public health, Wolaita Sodao University, Sodo, Ethiopia; 4 Department of Epidemiology, College of Health Sciences, Jimma University, Jimma, Ethiopia; 5 Discipline of Public Health, Faculty of Medicine, Nursing and Health Sciences, Flinders University, Adelaide, Australia

**Keywords:** Prevalence, anti-tuberculosis, non-adherence, cross-sectional survey, mixed method, qualitative method, Ethiopia

## Abstract

**Background:**

Evidence exists pointing out how non-adherence to treatment remains a major hurdle to efficient tuberculosis control in developing countries. Many tuberculosis (Tb) patients do not complete their six-month course of anti-tuberculosis medications and are not aware of the importance of sputum re-examinations, thereby putting themselves at risk of developing multidrug-resistant and extensively drug-resistant forms of tuberculosis and relapse. However, there is a dearth of publications about non-adherence towards anti-Tb medication in these settings. We assessed the prevalence of and associated factors for anti-Tb treatment non-adherence in public health care facilities of South Ethiopia.

**Methods:**

This was a cross-sectional survey using both quantitative and qualitative methods. The quantitative study was conducted among 261 Tb patients from 17 health centers and one general hospital. The qualitative aspect included an in-depth interview of 14 key informants. For quantitative data, the analysis of descriptive statistics, bivariate and multiple logistic regression was carried out, while thematic framework analysis was applied for the qualitative data.

**Results:**

The prevalence of non-adherence towards anti-Tb treatment was 24.5%. Multiple logistic regression analysis demonstrated that poor knowledge towards tuberculosis and its treatment (AOR = 4.6, 95%CI: 1.4-15.6), cost of medication other than Tb (AOR = 4.7, 95%CI: 1.7-13.4), having of health information at every visit (AOR = 3, 95% CI: 1.1-8.4) and distance of DOTS center from individual home (AOR = 5.7, 95%CI: 1.9-16.8) showed statistically significant association with non-adherence towards anti- tuberculosis treatment. Qualitative study also revealed that distance, lack of awareness about importance of treatment completion and cost of transportation were the major barriers for adherence.

**Conclusions:**

A quarter of Tb patients interrupted their treatment due to knowledge, availability and accessibility of DOTS service. We recommend creating awareness about anti-Tb treatment, and decentralization of drug pick-ups to the lowest level of health institutions.

## Background

Evidence exists that tuberculosis (Tb) is a major public health problem throughout the world, with an estimated 9.6 million annual incident cases, of which 1.2 million (12%) were co-infected with HIV and 1.5 million died globally in 2014 [1]. South East Asia (29%) and Africa (26%) accounted for the highest number of Tb cases, and both continents also shared 75% of global Tb deaths [2]. India, china and South Africa are countries with the highest burden of Tb accounting for 1.98 million [3], 1.4 million [2] and 0.5 million [4] respectively.

Standard Tb treatment requires patients to take a complex combination of drugs for 2 months in intensive phase and 4 months in continuation phase for new patients [5]. In the treatment of patients with MDR-Tb, an intensive phase of at least 8 months and a total treatment duration of at least 24 months is recommended [5]. This leads to non-adherence towards anti-Tb treatment. Directly observed therapy short course (DOTS), the use of the most effective standardized, short-course regimen, and of fixed-dose drug combinations (FDCs) under observation of health workers or family members, was the key strategy to improve adherence to anti-Tb treatment [5]. Ethiopia subscribed to the internationally accepted World Health Organization (WHO) strategy for Tb control and the DOTS programme was implemented since 1997 [5]. Nevertheless, coverage of DOTS service was still minimal and made little progress on treatment adherence in some resource- meager countries such as Ethiopia [6–8].

Even with over 80 million dollar USD funding from international agencies, and with counterpart funding from the Ethiopian government annually, Tb control remains modest [9]. Ethiopia, ranked 8th place, had 261 incident and 394 prevalent cases per 100,000 populations in 2010 [10]. A prevalence rate of 28.6% Tb/HIV co-infection [8] and 15.3% MDR-Tb was reported in the country [11]. Non-adherence to Tb treatment was well thought out an important barrier to Tb control [12]. It causes treatment failure and Tb relapse, and can further lead to prolonged infection, transmission, drug resistance and mortality [13].

In Ethiopia, different studies assessed the rates and determinants of anti-Tb non-adherence. For example, a study conducted North Ethiopia reported a prevalence rate of 11.5% [14]. Another study from Northwest Ethiopia reported a prevalence rate of 18.3% [15]. Being positive for human immunodeficiency virus (HIV), being on re-treatment for anti-Tb, knowledge about the disease, forgetfulness, social and economic factors played a role in patients’ refusal to comply with ant-Tb treatment [14–16]. However, most evidences come from retrospective surveys, few study settings, and solely from quantitative evidences. Thus, we assessed the prevalence of and factors associated for non-adherence towards anti-Tb treatment in 18 health care facilities in South Ethiopia using mixed methods.

## Methods

### Study design, settings and participants

This was an institution based cross-sectional survey that mixed quantitative and qualitative methods. We followed the guidelines on programmatic management of tuberculosis in Ethiopia for diagnosis and classification of Tb cases [5]. The zone is locate in Southern Nation, Nationalities and Peoples Region (SNNPR) and is 500 km far from Addis Ababa, Capital city of Ethiopia, and has a projected total population of 582,552. In 2009, about 24, 554 all form of TB cases were registered in SNNPR. It has one general hospital, 21 health centers, 175 health posts and 404 health professionals. The potential health coverage i.e. access to health services when needed and avoid financial hardship in paying for those services of the zone is 91%. DOTs service is provided in 17 health centers and one General hospital. Data were collected from all these health facilities: Tercha General Hospital, Bale health center (HC), Dali HC, Hagle HC, Tocha HC, Mari HC, Waka HC, Gessa HC, Loma HC, Yello HC, Wara HC, Wh HC, Kechi HC, Boka HC, Kerwo HC, Duga HC, Dash HC and Gendo HC between February and April 2013.

The study population included pulmonary Tb patients who registered 6 months before data collection time on anti-Tb treatment regime. Patients on MDR treatment regimen and patients too ill to be interviewed were excluded from the study. The sample size was calculated via OpenEpi 2.3 software using a single population proportion calculation formula using the following assumptions: 20% proportion of non adherence towards ant-Tb treatment [17], 95% confidence level, 5% margin of error and 10% non-response rate. The total calculated sample yielded 271. Using sampling frame of records, simple random sampling technique was used to recruit the study participants. For qualitative data, 14 individuals (7 healthcare providers, 4 Tb patients and 3 health extension workers) were selected purposefully from the hospital and health centers.

### Variables in the study and its measurement

Anti-Tb treatment adherence status was the response variable dichotomized as adherent and non adherent. A patient belonging to either intensive or continuation phase under new or retreatment regimen who missed ≥10% of the total prescribed dose was considered as non-adherent. For sputum follow up, patient who missed one and more sputum test will be considered as non-adherent. The covariates included: sex, age, religion, ethnicity, level of education, occupation, marital status, having treatment supporter, type of transportation, attitude of care providers, alcohol consumption, cigarette smoking, having patient supervisor, availability of health information at every visit, side effect of medication, current treatment phase, pill burden, knowledge about Tb and its treatment, distance from DOT center, traveling time and Cost of medication other than anti-Tb.

History of smoking and history of alcohol consumption has been assessed as during lifetime. We asked participants to report their lifetime experiences of cigarette smoking and alcohol drinking. We recorded their response as smoker, non-smoker and ex-smoker for cigarette smoking; drinker or non- drinker for alcohol drinking. For statistical analysis, smoking responses was further aggregated to yes or no. Knowledge towards Tb and its treatment was assessed using knowledge related questions and was classified as poor, fair and good. Scores ≥75%, 74-60% and <60%, respectively, were labeled as good, fair and poor knowledge.

### Data collection

Face-to-face interviews were conducted by six registered nurses and supervised by three health officers to solicit socio-demographic and economic factors, treatment related factors, behavioral factors and patient knowledge towards tuberculosis and its treatment. The interviews were conducted in a quiet room at the Tb clinic where patients came for follow up checkup. Study subjects who lost from follow-up were interviewed at home. Qualitative data were collated through notes and audio-recordings during interview. We used in-depth interview guide to explore barriers related with current Tb control programs and possible reasons for treatment non-adherence.

### Statistical analysis

Quantitative data were analyzed using descriptive and inferential statistical techniques. Summary statistics including percentages and odds ratios were computed. We used binary logistic regression to analyze factors that were associated with adherence to anti-Tb medications. The analyses of bivariate logistic regression assessment were conducted to check the existence of crude association and select the candidate variables (*P* < 0.25 was considered).

We checked multi-collinearity among selected independent variables via variance inflation factor (VIF) and none was found. Multiple logistic regression analysis was used to declare the independently associated predictors. *P*-value ≤0.05 was considered as a cut off point for statistical significance in the final model. Goodness of fit of the final model was checked by Hosmer and Lemeshow [18] and was found fit. The Data were summarized using odds ratio (OR) and 95% confidence interval. All analyses were conducted in Statistical Package for the Social Sciences (SPSS) version 16.0 for windows [19].

Qualitative data were analysed using thematic framework analysis [20–22]. We conducted the following six steps: i) Transcriptions were carried out from the audiotaped interviews; ii) All transcripts were repeatedly read by all authors to familiarize with the data; iii) After careful reading, codes were developed so that similar codes would be grouped together into categories: the analysis was both deductive and inductive type to establish codes known apriority and newly emerged from the data; iv) Working analytical framework was developed while grouping similar codes into categories; v) data were summarized by category from each transcript using a matrix spreadsheet, a process called charting; vi) interpreting the data was the last step. Findings from patients, health professionals and health extension workers were triangulated. This helps to the validity of the data and show conflicting views.

## Results

### Socio-demographic and clinical characteristics of respondents

A total of 261 (96.3%) Tb patients were considered eligible: 36 patients from hospital and 225 from health centers. Data of 10 study subjects were not included in the analysis due to changing of place of residence, death and incompleteness. Table 1 shows demographic and clinical profile of the respondents. Males were slightly over-represented (53.6%) and almost one third (32.6%) of the respondents represented youths aged between 15 and 24 years. Half (50.2%) of the respondents followed orthodox Christian and two third (65%) of the respondents were married. Two out of five (43.9%) respondents didn’t attend a school, and about 33% of respondents were housewives. Majority (60.2%) of the patients were pulmonary Tb positive, and almost all (98%) were categorized as new patients. Nearly two-third of patients were in the intensive phase of Tb treatment. Prevalence of Tb/HIV co-infection in the current study was 3.1%.

**Table 1:** Demographic and clinical characteristics of pulmonary TB patients in Dawouro Zone public health care facilities, South West Ethiopia, 2012/2013

Characteristics	Number, n	Percent
Sex		
Male	140	53.6
Female	121	46.4
Age (years)		
15-24	85	32.6
25-34	80	30.7
35-44	54	20.7
≥ 45	42	16.0
Marital status		
Married	170	65.0
Single	80	30.7
Widowed	7	2.7
Divorced	3	1.2
Separated	1	0.4
Religions		
Orthodox	131	50.2
Protestants	125	47.9
Catholic	5	1.9
Ethnicity		
Dawuro	*245*	93.9
Wolayta	10	3.8
Others ^a^	6	2.3
Educational status		
Illiterate	112	42.9
Able only to read/write	26	10.0
First cycle	58	22.2
Second cycle	36	13.8
Preparatory	7	2.7
College/university	22	8.4
Occupational status		
Housewife	85	32.6
Farmer	79	30.3
Student	57	21.8
Government employee	31	11.9
Others ^b^	9	3.4
Disease classification		
Pulmonary TB positive	157	60.2
Pulmonary TB negative	104	39.8
Patients category		
New	256	98.0
Treatment failure	2	0.8
Relapse	2	0.8
Return after default	1	0.4
Treatment phase		
Intensive phase	163	62.5
Continuation phase	98	37.5
Treatment regimen		
New patient treatment regimen	256	98.1
Retreatment patient regimen	5	1.9
HIV status		
Sero-negative	232	88.9
Sero- positive	8	3.1
Unspecified	21	8.0

^a^ Amhara, Wolayta, Kambata, Hadeya; ^b^ Merchants, house maid

### Prevalence of anti-Tb non adherence

The prevalence of anti-Tb non-adherence was 24.5%. Among the total non-adherents, 36 were males, 36 were pulmonary Tb positive, and 32 were under intensive phase when analyzed by sex, Tb classification and treatment phase respectively.

### Barriers of anti-Tb treatment adherence among pulmonary Tb patients

Table 2 presents findings of bivariate logistic regression analysis between socio-demographic and economic factors and non-adherence towards anti-Tb medication. Table 3 presents bivariate logistic regression analysis of clinical factors with non-adherence towards anti-Tb medication. According to the bivariate logistic regression analyses, level of education and occupational status were the only statistically significant demographic variables. Type of transportation, attitude of health care providers, having a patient supervisor, availability of health information at every visit, side effect of medication, current treatment phase, pill burden, knowledge, cost of medication other than anti-Tb, distance and travelling time were statistically significant clinical variables.

**Table 2:** Bivarate analysis of socio-demographic and economic factors of treatment non-adherence of pulmonary TB patients in public health care facilities in Dawuro zone, Southwest Ethiopia, 2012/2013

Variables	Adherents Numbers (%)	Non-adherents Numbers (%)	COR (95%CI)	*P* value
Sex				
Female	93(76.9)	28(23.1)	1	
Male	104(74.3)	36(25.7)	1.2(0.7-2.0)	0.6
Age (years)				
15-24	67 (78.8)	18(21.2)	1	
25-34	60(75.0)	20(25.0)	1.2(0.6-2.6)	0.6
35-44	39(72.2)	15(27.8)	1.4(0.7-3.2)	0.4
≥ 45	31(73.8)	11(26.2)	1.3(0.6-3.1)	0.5
Marital status				
Single	63(78.8)	17(21.2)	1	
Married	126(74.1)	44(25.9)	1.3(0.7-2.5)	0.4
Divorced& widowed	8(72.7)	3(27.3)	1.4(0.3-5.8)	0.7
Religions				
Orthodox	100(76.3)	31(23.7)	1	
Others ^a^	97(74.6)	33(25.4)	1.1(0.6-1.9)	0.8
Ethnicity				
Dawouro	186(75.9)	59(24.1)	1	
Others ^b^	11(68.8)	5(31.2)	1.4(0.5-4.3)	0.5
Educational status				
Formal education	101(82.1)	22(17.9)	1	
Non-formal education	96(69.6)	42(30.4)	2.1(1.1-3.6)	0.02*
Occupational status				
Gov. employee	28(90.3)	3(9.7)	1	
Housewife	62(72.9)	23(27.1)	3.5(0.9-12.3)	0.05*
Farmers	52(65.8)	27(34.2)	4.9(1.4-17.3)	0.02*
Others ^c^	55(83.3)	11(16.7)	1.9(0.5-7.2)	0.4

*AOR* adjusted odds ratio, *COR* crude odds ratio, *CI* confidence interval

* Statistically significant at *P*-value below 0.05

^a^ Protestant, Catholic; ^b^ Amhara, Wolayta, Kambata, Hadeya; ^c^ Merchants, house maid

**Table 3:** Bivarate analysis of clinical predictors of anti-TB treatment non-adherence of pulmonary TB patients in public health care facilities, Dawuro Zone, South west Ethiopia, 2012/2013

Variables	Adherents No (%)	Non-adherents No (%)	COR (95%CI)	*P* Value
Having treatment supporter				
Yes	181(77.0)	54(23.0)	1	
No	16(61.5)	10(38.5)	2.1(1.0-4.9)	0.087
Type of transportation				
Walking	173(83.2)	35(16.8)	1	
Use own transport	11(44.0)	14(56.0)	6.3(2.6-15.0)	<0.001*
Use public transport	11(42.3)	15(57.7)	6.7(2.9-15.9)	<0.001
Attitude of care providers				
Care friendly	180(78.3)	50(21.7)	1	
Care unfriendly	17(54.8)	14(45.2)	3.0(1.4-6.4)	0.01*
Alcohol consumption				
No	186(76.9)	56(23.1)	1	
Yes	11(57.9)	8(42.1)	2.4(0.9-6.3)	0.07
Having patient supervisor				
Yes	177(77.0)	53(23.0)	1	
No	5(35.7)	9(64.3)	6.0(1.9-18.7)	0.002*
Getting health education at every visit				
Yes	130(88.4)	17(11.6)	1	
No	67(58.8)	47(41.2)	5.4(2.9-10.1)	<0.001*
Medication side effect				
Absent	176(77.9)	50(22.1)	1	
Presents	21(60.0)	14(40.0)	2.3(1.1-4.9)	0.025*
Current treatment phase				
Continuation	134(80.7)	32(19.3)	1	
Intensive	63(66.3)	32(33.7)	2.1(1.2-3.8)	0.01*
Pill burden				
Absent	190(76.9)	57(23.1)	1	
Present	7(50.0)	7(50.0)	3.3(1.1-9.9)	0.03*
Knowledge				
Good	107(89.9)	12(10.1)	1	
Fair	41(87.2)	6(12.8)	1.3(0.5-3.7)	0.09
Poor	49(51.6)	46(48.4)	8.4(4.1-17.2)	<0.001*
Distance of DOT center				
< 10 km	171(85.5)	29(14.5)	1	
≥ 10 km	26(42.6)	35(57.4)	7.9(4.2-15.1)	<0.001*
Traveling time				
< 60 min	122(86.5)	19(19.5)	1	
≥ 60 min	75(62.5)	45(37.5)	3.9(2.1-7.1)	<0.001*
Cost of medication other than anti-Tb				
No	143(92.9)	11(7.1)	1	
Yes	54(50.5)	53(49.5)	12.7(6.2-26.2)	<0.001*

*AOR* adjusted odds ratio, *COR* crude odds ratio, *CI* confidence interval

* Statistically significant at *P*-value below 0.05

Table 4 presents the multiple logistic regression analysis with non-adherence towards anti-Tb medication. Logistic regression analyses demonstrated the following factors statistically associated with non-adherence: having poor level of knowledge, absence of health information at every visit, pill burden, distance to Tb clinic beyond 10 km, and cost of medication other than anti-Tb.

**Table 4:** Multivariable Logistic regression model to identify independent predictor’s of treatment non-adherence of pulmonary TB patients in public health care facilities in Dawuro Zone, South west Ethiopia, 2012/2013

Variables	Adherents No (%)	Non-adherent No (%)	COR (95%CI)	AOR (95%CI)
Educational status				
Formal education	101(82.1)	22(17.9)	1	1
Non-formal education	96(69.6)	42(30.4)	2.1(1.1-3.6)	0.6(0.2-2.4)
Occupational status				
Gov. employee	28(90.3)	3(9.7)	1	1
Housewife	62(72.9)	23(27.1)	3.5(0.9-12.3)	1.8(0.3-11.9)
Farmers	52(65.8)	27(34.2)	4.9(1.4-17.3)	1.7(0.2-19.6)
Others ^a^	55(83.3)	11(16.7)	1.9(0.5-7.2)	1.3(0.2-7.2)
Treatment supporter				
Present	181(77.0)	54(23.0)	1	1
Absent	16(61.5)	10(38.5)	2.1(1.0-4.9)	2.8(0.6-12.3)
Knowledge				
Good	107(89.9)	12(10.1)	1	1
Fair	41(87.2)	6(12.8)	1.3(0.5-3.7)	3.8(0.7-20.4)
Poor	49(51.6)	46(48.4)	8.4(4.1-17.2)	4.6(1.4-15.6)*
Attitude of care providers				
Care friendly	180(78.3)	50(21.7)	1	1
Care unfriendly	17(54.8)	14(45.2)	3.0(1.4-6.4)	1.2(0.3-5.9)
Traveling time				
< 60 min	122(86.5)	19(19.5)	1	1
≥ 60 min	75(62.5)	45(37.5)	3.9(2.1-7.1)	1.7(0.5-5.1)
Patient taking alcohol				
No	186(76.9)	56(23.1)	1	1
Yes	11(57.9)	8(42.1)	2.4(0.9-6.3)	1.2(0.1-11.5)
Patient has supervisor				
Yes	177(77.0)	53(23.0)	1	1
No	5(35.7)	9(64.3)	6.0(1.9-18.7)	0.5(0.04-5.9)
Health information at every visit				
Yes	130(88.4)	17(11.6)	1	1
No	67(58.8)	47(41.2)	5.4(2.9-10.1)	3.0(1.1-8.4)*
Medication side effect				
Absent	176(77.9)	50(22.1)	1	1
Presents	21(60.0)	14(40.0)	2.3(1.1-4.9)	1.7(0.4-7.1)
Current treatment phase				
Continuation	134(80.7)	32(19.3)	1	1
Intensive	63(66.3)	32(33.7)	2.1(1.2-3.8)	1.7(0.6-4.5)
Pill burden				
Absent	190(76.9)	57(23.1)	1	1
Present	7(50.0)	7(50.0)	3.3(1.1-9.9)	6.1(1.0-36.9)*
Distance of DOT center				
< 10 km	171(85.5)	29(14.5)	1	1
≥ 10 km	26(42.6)	35(57.4)	7.9(4.2-15.1)	5.7(1.9-16.8)*
Cost of medication other than anti-Tb				
No	143(92.9)	11(7.1)	1	1
Yes	54(50.5)	53(49.5)	12.7(6.2-26.2)	4.7(1.7-13.4)*
Type of transportation				
Walking	173(83.2)	35(16.8)	1	1
Use own transport	11(44.0)	14(56.0)	6.3(2.6-15.0)	2.7(0.5-15.0)
Use public transport	11(42.3)	15(57.7)	6.7(2.9-15.9)	4.8(0.9-25.0)

* Statistically significant at *P*-value below 0.05

^a^ Merchants, house maid

Tb patients who had poor knowledge about Tb and its treatment were 5 times (AOR = 4.6, 95%CI: 1.4-15.6) higher at risk towards anti-Tb non-adherence than those who had good knowledge. The relative probability of anti-Tb non-adherence among patients travelled more than 10 km to pick treatments was higher than (AOR = 5.7, 95% CI: 1.9–16.8) those travelled less than 10 km. The association of anti-Tb non-adherence among patients who didn’t get health information at every visit was 3 times (AOR = 3; 95% CI: 1.1–8.4) higher than among those who got. Cost of medication other than Tb was found a risk factor (AOR = 4.7, 95%CI: 1.7-13.4) to anti-Tb non-adherence.

Findings of the qualitative study also supported the quantitative results. The respondents stated that there was an improvement of the program. However, they reported that the service is not decentralized, lack of drugs in stock was still observed and shortage of laboratory reagents for AFB was becoming a routine challenge. Distance, lack of awareness about importance of treatment completion and cost of transportation were the explored barriers for adherence. Decentralization of drug pick-ups and delivering health information at every visit were the suggested solutions. Table 5 presents some of the quotes responded by the participants.

**Table 5:** Examples of interview extracts of Tb patients, health extension workers and health professionals concerning barriers for anti-Tb non-adherence

Barriers for non-adherence	Patient and health professionals citations
Distance and travelling cost	“*Against to the national tuberculosis guide line, we give at least two days medication to their home during intensive phase…. to decrease the distance burden and traveling cost”* TB clinic nurse from health center “*Treatment adherence of patient in our facilities is relatively improving but majorities of patients do not return for sputum re-examination specially for 5th and 6th month check up due to distance”* TB clinic nurse from hospital
Drug pick-ups decentralization	“*To improve the patient’s adherence health post should provide the DOTS service. Health extension workers are providing family planning service including injectable, so why not DOTS service?”* Health extension worker
Distance, cost of transportation and DOTS service decentralization	“*Patients like me, who come from long distance can’t complete the treatment even if we have enough money. Because, we have another business in our home. But if the service is near to my home, I know I can complete the treatment easily. For example our health post is very close to me. Due to the absence of this access, we are forced to travel this long distance and pay more than 40 ETB daily.”* Patient from health center

## Discussion

Adherence to TB treatment is fundamental for successful TB control and eradication. Nonetheless, the long-term course of the treatment might expose patients for non-adherence [23]. In the current study, only 75.5% of participants were able to adhere to anti-Tb regimen, which was lower than the reported adherence rates in North East Ethiopia (77.5) [14] and Southwest Ethiopia (79.2%) [24]. We argue that, these differences in findings could be due to variations in definitions of anti-Tb non-adherence. No gold standard definition of adherence towards ant-Tb treatment is yet available although quantity and timing of missed medication or hospital appointments were recommended by WHO. In our study, patients who missed 10% or more of their prescribed doses of anti-Tb drugs were considered as non-adherent while others measured non-adherence if patients discontinued medication for 6 days [25, 26].

In the current study, knowledge, distance, health information at every visit, pill burden and cost of medication other than anti-Tb were the predictors for anti-Tb treatment non-adherence. Patients who had poor knowledge towards Tb and its treatment were at risk of being non-adherent. This was suggested by the qualitative findings of the current study. The result was corroborated by findings of previous studies [27–29]. This might plausibly be justified by that majority of the respondents were also illiterate. This cues an action to increase awareness about the disease and its treatment. It is well known that education increases knowledge, health awareness and treatment seeking behavior of individuals [30]. According to both quantitative and qualitative findings of the current study, distance was another barrier for anti-Tb treatment non-adherence, a finding that was similarly reported by studies done elsewhere [15, 28, 31]. This heed the need for decentralization of DOTs service to health post- the lowest level of health facility structure in Ethiopia.

Cost of medication other than anti-Tb remains another serious challenge in Tb treatment non-adherence. This was not dissimilar with the findings of previous studies [30, 32]. This is because Ethiopia’s national Tb program requires free provision of anti-Tb treatment and its examination [5, 33]. Nonetheless, this free Tb service policy did not include drugs other than anti-Tb. For example, drugs related to respiratory symptoms, liver protection, or adverse effects associated with anti-Tb drugs are not covered. In addition, patients worry transportation and accommodation costs. Decentralization of drug pick-ups might reduce the burden. Health care financing progam should also consider Tb patients to waive costs associated with adverse reactions of anti-Tb drugs.

The study had some limitations, which were worth noting. The institutional based nature of the study might not infer for other Tb patients who didn’t visit the institution. Similarly, the nature of cross-sectional study design does not indicate temporal relationship or causality. Self-report of adherence to medications could also be affected by recall bias. Moreover, selection bias could also have been introduced because patients who are under regular follow-up by the university clinic are likely to be receiving better care and support than those in the lower level clinics. Health workers collected data through direct interview of patients and this may subject to social desirability bias.

## Conclusions

In conclusion, this current study assessed the degree of adherence in pulmonary Tb patients receiving DOTS therapy and barriers contributing to adherence. In comparison to previous studies, our study revealed relatively high non-adherence rate among new pulmonary Tb patients. Knowledge, distance, not decentralizing of DOTs service, health information at every visit and cost of medication other than anti-Tb were the barriers for anti-Tb treatment adherence. Policies are recommended to be developed to strengthen the treatment management of TB patients. Where possible, decentralization of DOTs service to lower level of health facility structure should be prioritized.

## Abbreviations

CI: Confidence interval
CPT: Co-trimoxazole preventive therapy
DOTS: Directly observed treatment short-course
HIV: Human immunodeficiency virus
MDR: Multi drug resistance
NTLCP: National Tuberculosis and Leprosy Control Program
SNNPR: South Nation Nationality Peoples Region
Tb: Tuberculosis
VIF: Variance inflation factor
WHO: World Health Organization

## References

[1] WHO. Global tuberculosis report 2015. Geneva: 2015

[2] WHO. Global tuberculosis report 2013. Geneva: 2013

[3] Shekhar M, Minal Vachali M, Mansi C, Madhukar P: TB Diagnostics in India: Market Analysis and Potential Revised - Version 2. Montreal: WHO; 2012.

[4] Kanabus A: TB Statistics for South Africa – National & provincial. In. vol. 2016; 2016. [http://www.tbfacts.org/tb-statistics-south-africa/].

[5] FDRE, MOH. Guidelines On Programmatic Management Of Drug Resistant Tuberculosis In Ethiopia: 2012. Addis Ababa: Ministry of Health of Ethiopia; 2012.

[6] Shargie EB, Lindtjørn B. DOTS improves treatment outcomes and service coverage for tuberculosis in South Ethiopia: A retrospective trend analysis. BMC Public Health. 2005;5 10.1186/1471-2458-5-62 PMC1173119 15938746

[7] Garrido MS, Penna ML, Perez-Porcuna TM, ABd S, LdS M, Albuquerque BC, FE MÌ-E, Bührer-Sékula S (2012). Factors Associated with Tuberculosis Treatment Default in an Endemic Area of the Brazilian Amazon: A Case Control-Study. PLoS One.

[8] Volmink J, Garner P: Directly observed therapy for treating tuberculosis. The Cochrane database of systematic reviews 2007;17(4):Cd003343. 10.1002/14651858.CD003343.pub3 17943789

[9] WHO. Estimates of TB and MDR-TB burden. Geneva: 2016.

[10] WHO 2011 report on global tuberculosis control. Geneva: WHO; 2011.

[11] Nigus DMLW, Beyene BA, Tamiru AA, Lemma MT (2014). Prevalence of Multi Drug Resistant Tuberculosis among Presumptive Multi Drug Resistant Tuberculosis Cases in Amhara National Regional State, Ethiopia. J Mycobac Dis.

[12] Munro SA, Lewin SA, Smith HJ, Engel ME, Fretheim A, Volmink J (2007). Patient Adherence to Tuberculosis Treatment: A Systematic Review of Qualitative Research. PLoS Med.

[13] Maartens G, Wilkinson RJ (2007). Tuberculosis. Lancet.

[14] Tesfahuneygn G, Medhin G, Legesse M (2015). Adherence to Anti-tuberculosis treatment and treatment outcomes among tuberculosis patients in Alamata District, northeast Ethiopia. BMC. Res. Notes.

[15] Bagchi S, Ambe G, Sathiakumar N (2010). Determinants of poor adherence to anti-tuberculosis treatment in mumbai, India. Int J. Prev. Med.

[16] Adane AA, Alene KA, Koye DN, Zeleke BM (2013). Non-Adherence to Anti-Tuberculosis Treatment and Determinant Factors among Patients with Tuberculosis in Northwest Ethiopia. PLoS One.

[17] Shargie E, B L (2007). Determinants of treatment adherence among smear-positive pulmonary tuberculosis patients in Southern Ethiopia. PLoS Med.

[18] Hosmer DW, Hosmer T, Le Cessie S, Lemeshow S (1997). A comparison of goodness-of-fit tests for the logistic regression model. Stat Med.

[19] SPSS Inc. Released 2007. SPSS for Windows, Version 16.0. Chicago, SPSS Inc. In.

[20] Gale NK, Heath G, Cameron E, Rashid S, Redwood S (2013). Using the framework method for the analysis of qualitative data in multi-disciplinary health research. BMC Med Res Methodol.

[21] Srivastava A, Thomson SB (2009). Framework Analysis: A Qualitative Methodology for Applied Policy Research. JOAAG.

[22] Ritchie Jane SL (2002). Qualitative Data Analysis for Applied Policy Research. The Qualitative Researcher's Companion. SAGE Publications, Inc.

[23] WHO. Adherence to long-term therapies: evidence for action. Geneva: 2003.

[24] Kebede A, Wabe NT (2012). Medication adherence and its determinants among patients on concomitant tuberculosis and antiretroviral therapy in South west ethiopia. N Am J Med Sci.

[25] Hu D, Liu X, Chen J, Wang Y, Wang T, Zeng W, Smith H, Garner P (2008). Direct observation and adherence to tuberculosis treatment in Chongqing, China: a descriptive study. Health Policy Plan.

[26] Xu W, Lu W, Zhou Y, Zhu L, Shen H, Wang J (2009). Adherence to anti-tuberculosis treatment among pulmonary tuberculosis patients: a qualitative and quantitative study. BMC Health Serv Res.

[27] Kulkarni PY, Akarte SV, Mankeshwar RM, Bhawalkar JS, Banerjee A, Kulkarni AD (2013). Non-Adherence of New Pulmonary Tuberculosis Patients to Anti-Tuberculosis Treatment. Ann Medical Health Sci Res.

[28] Castelnuovo B (2010). A review of compliance to anti tuberculosis treatment and risk factors for defaulting treatment in Sub Saharan Africa. Afr Health Sci.

[29] Tang Y, Zhao M, Wang Y, Gong Y, Yin X, Zhao A, Zheng J, Liu Z, Jian X, Wang W (2015). Non-adherence to anti-tuberculosis treatment among internal migrants with pulmonary tuberculosis in Shenzhen, China: a cross-sectional study. BMC Public Health.

[30] Lei X, Huang K, Liu Q, Jie YF, Tang SL (2016). Are tuberculosis patients adherent to prescribed treatments in China? Results of a prospective cohort study. Infect Dis Poverty.

[31] Tola HH, Tol A, Shojaeizadeh D, Garmaroudi G (2015). Tuberculosis treatment non-adherence and lost to follow up among TB patients with or without HIV in developing countries: A systematic review. Iranian J Public Health.

[32] Long Q, Smith H, Zhang T, Tang S, Garner P (2011). Patient medical costs for tuberculosis treatment and impact on adherence in China: a systematic review. BMC Public Health.

[33] FDRE (2010). Health Sector Development Program IV 2010/11 – 2014/15. vol. Final Draft.

